# Beneficial Effect of Ultra-Low-Dose Aspirin in Platelet Activity Alterations and Haemorrhage Observed in Experimental Portal Hypertension

**DOI:** 10.1155/2012/430460

**Published:** 2011-11-14

**Authors:** F. X. Eizayaga, O. Aguejouf, V. Desplat, C. Doutremepuich

**Affiliations:** Laboratoire d'Hématologie, UFR des Sciences Pharmaceutiques, Université de Bordeaux 2, Victor Segalen, 33076 Bordeaux Cedex, France

## Abstract

Ultra-low-dose aspirin has shown a prothrombotic effect in the laser-induced thrombosis model. Several studies of our laboratory have shown a positive effect in rats with two different experimental models of portal hypertension: portal vein ligation, a model with an almost normal liver, and 30 days of bile duct ligation, a model with cirrhosis and presence of ascitis. In both models of portal hypertensive rats, bleeding time was prolonged and thrombi formation, in a laser-induced model of thrombi production, decreased. The hypotheses of the presented studies were that ultra-low-dose aspirin could decrease the bleeding complications in these models and that the mechanism for these effects could act thorough the COX pathway. In different studies, ultra-low dose of aspirin normalized the induced hemorrhage time, thrombi production, and platelet-endothelial cell interaction. The possible beneficial role of these doses of aspirin and mechanism of COX 2 inhibition are discussed.

## 1. Introduction

Hemorrhage in portal hypertension is still a lethal complication of cirrhosis in patients in whom clinical decompensation has already developed. Treatment of hemorrhage risk is pointed to the decrease of elevated portal pressure, mostly by vasoconstrictors, and in some cases to the decrease of elevated liver increased vascular resistance [1]. However, the cause of hemorrhage increased risk is multifactorial. Primary and secondary haemostases as well as fibrinolysis are altered [2]. 

Primary haemostasis alterations are an important component of the haemorrhage observed in hepatic cirrhosis and were firstly described by Thomas et al. as alterations of platelet aggregation [3, 4]. Since then, multiple platelet problems have been described: disorders of prostanoid synthesis, defective signal transduction, defects in platelet glycoprotein Ib, and a storage pool defect [5–8]. Platelet adhesion, the first step in platelet function following endothelial damage, is also altered in liver cirrhosis. Although the nature of platelet alterations is multifactorial, the impairment in platelet adhesion was the more evident finding in cirrhotic patients, even those with compensated cirrhosis in a study of Ordinas et al. [9]. The method described by this group, which studies platelet adhesion under flow conditions, shows platelet adhesion impairments present in cirrhotic patients that are more consistent than the changes found with standard aggregometric procedures. Increased endothelial synthesis of potent inhibitors of platelet aggregation, nitric oxide (NO), and prostacyclin (PGI_2_) also takes part in the impairment of primary haemostasis present in hepatic cirrhosis. In a previous study done by Albornoz et al., platelet adhesion and haemorrhagic time were normalized after inhibiting NO synthesis with N(G)-nitro-L-arginine (LNNA) in bile-duct-ligated rats [10]. Despite these studies, the importance of platelet dysfunction to the haemostatic disturbance in cirrhosis has not been completely elucidated nor treatments of hemorrhage in portal hypertension aimed to correct these problems. 

Ultra-low-dose aspirin produces an increased interaction between platelets and endothelial cells in the normal rat. Portal hypertension produced a decreased interaction between platelets and endothelial cells and a prolonged hemorrhagic time. These interaction alterations as well as hemorrhage have been shown to be normalized in experimental portal hypertension models in the rat. In this paper the effects of ultra-low-dose aspirin in rats with portal hypertension and the mechanism underlying this effect will be reviewed.

## 2. Methods

### 2.1. Animals

Male Wistar rats (200–250 g) purchased from Delpre Breeding Center (St. Doulchard, France) were housed separately and acclimatized before use under conditions of controlled temperature (25 ± 2°C) and illumination (12 h light/dark cycle). They were fed with standard rat chow and water *ad libitum*. Animals received care in compliance with the European Convention of Animal Care.

### 2.2. Surgical Procedures

#### 2.2.1. Production of Portal Hypertension

After 1 week of acclimatization, rats were randomized and separated in two groups: one consisted in sham-operated rats and the other formed by portal hypertensive rats. Portal hypertension was induced by a calibrated portal vein stenosis, according to the procedure described by Vorobioff et al. [11]. Rats were anesthetized with Ketamine (Panpharma, Fougères, France) 90 mg/kg body weight, i.m., and then a midline abdominal incision was made. The portal vein was located and isolated from the surrounding tissues. A ligature of 3-0 silk was placed around the vein and snugly tied to a 20 gauge blunt-end needle placed alongside the portal vein. The needle was subsequently removed to yield a calibrated stenosis of the portal vein. Sham-operated rats underwent an identical procedure except that portal vein was isolated but not stenosed.

Animals were housed during fourteen days after the operation to develop portal hypertension in the corresponding group.

#### 2.2.2. Production of Biliary Cirrhosis

Cirrhosis was produced by bile duct ligation (BDL), similar to the procedure described by Kountouras et al. [12]. Rats were anesthetized with Ketamine (Panpharma, Fougères, France) 90 mg/kg body weight, i.m., and then a midline abdominal incision was made. In the BDL group, the common bile duct was isolated, double-ligated with nonresorbable suture (silk 3-0), and up to a 6-mm section resected between the two ligatures. The abdominal incision was then closed with sutures (Vycril 4.0), and the rats were allowed to recover. In the control group, the abdomen was closed following minimal manipulation of the abdominal content. BDL and control rats were then studied thirty days after surgery.

### 2.3. Thrombus Induction

Animals were anesthetized with 200 mg/kg of thiopental sodium (Pentothal, Laboratoires Abbott, Rungis, France), and a median laparotomy was performed. The intestinal loop was placed on the microscope table, and vascular lesions were induced by Argon laser (Stabilite 2016, Spectra Physics, France). 

The wavelength used was 514 nm, and the energy was adjusted to 120 mW. The laser beam was applied for 1/15 sec. The dynamic course of thrombus formation was continuously monitored with an inverted microscope (Axiovert, Zeiss, France). A schematic view of the apparatus used has been previously described [13]. Arterioles between 15 and 25 *μ*m diameter were used. 

Two parameters were assessed during each procedure: the number of emboli (NE) removed from the thrombus by blood flow after an injury produced by the laser shot and the duration of embolization (DE), defined as the time between the first and the last emboli occurring after thrombus formation, expressed in minutes.

### 2.4. Induced Hemorrhagic Time

An experimental model of induced hemorrhagic time (IHT) was performed 10 minutes before thrombosis induction by laser. The tail of the rat was immersed in water for 5 minutes at 37°C and sectioned 6 mm from the extremity. IHT measured corresponded to the time between the tail section and the end of bleeding, expressed in seconds.

### 2.5. Drugs Tested

The amounts of 1 mg/mL and 100 mg/mL were obtained by diluting a solution of acetylsalicylate (Aspegic, Sanofi-synthelabo, France) 500 mg/5mL. Aspirin dilutions were purchased from Boiron Laboratories (Sainte-Foy-Les-Lyon, France) and were prepared as follows. 1 g of pure, finely powdered aspirin were suspended in 99 mL of alcohol (70°). After being vigorously shaken, 1 mL of this dilution was then mixed with 99 mL of distilled water and vigorously shaken (dilution 1). The latter process was repeated until obtaining desired dilutions 14 times (dilution 15). Alcohol and sterilized water following the above-mentioned procedures without adding the aspirin was used as placebo of dilution 15. Aspirin or the corresponding placebo was subcutaneously administered at a final volume of 1 mL/kg rat weight. The different placebos were used to avoid interferences due to the different kinds of preparations of aspirin used. Dilution 15 of aspirin was reported to have prothrombotic effect in previous studies [13].

Indomethacin (Indocid, MSD, Merck, Paris, France) and NAME (nitro-arginine-methyl ester, Sigma Aldrich, Saint Quentin Fallavier, France) were injected subcutaneously at doses of 2.5 mg/kg and 30 mg/kg, respectively, prepared in a final volume of 1 mL/kg of rat weight.

Selective inhibitors of COX 1, SC-560 and of COX 2, NS-398, were purchased from Cayman Chemical (Ann Arbor, Mich, USA), and suspended in carboxymethyl-cellulose (CMC) 0.5 g/L at a final volume of 1 mL/kg rat weight. The CMC solution without adding the inhibitors was used as placebo. COX selective inhibitors were used at the dose of 10 mg/kg and were administered *per os*. 

SC-560 is a member of the diaryl heterocycle class of COX inhibitors which includes celecoxib (Celebrex) and rofecoxib (Vioxx). However, unlike these selective COX 2 inhibitors, SC-560 is a selective inhibitor of COX 1. Using human recombinant enzymes, the IC50 value for SC-560 with respect to COX 1 is 9 nM, while the corresponding IC_50_ value for COX 2 is 6.3 *μ*M. Thus, SC-560 shows 700-fold selectivity for the COX 1 enzyme. SC-560 is orally active in the rat, where 10 mg/kg completely abolishes the ionophore-induced production of thromboxane B_2_ in whole blood [14–16]. 

NS-398 is a selective inhibitor of COX 2. The IC_50_ values for human recombinant COX 1 and 2 are 75 and 1.77 *μ*M, respectively. The IC_50_ values for ovine COX 1 and 2 are 220 and 0.15 *μ*M, respectively, [17, 18]. 

## 3. Results and Discussion

### 3.1. First Study

#### 3.1.1. Modifications of Laser-Induced Thrombosis and Hemorrhage Produced by Experimental Prehepatic Portal Hypertension. Effects of Ultra-Low-Dose Aspirin

The first study was done in our Laboratory with 4 groups of rats. Two of them underwent portal vein ligation surgery and developed portal hypertension. In the other two groups, the portal vein was identified but not ligated (sham-operated groups). One of the portal hypertensive groups received ultra-low-dose aspirin, and the other received placebo. The same was repeated with the sham-operated groups. Two separated studies were done to duplicate the observations. The first pilot study was done with an *n* = 5 to 9 rats per group and the confirmatory study with *n* = 25 to 32 rats per group. This study was published in 2005 [19]. After 2 weeks of portal vein ligation, portal hypertensive rats have shown decreased thrombi formation expressed as a decreased number of emboli and a decreased embolization time. Induced hemorrhagic time was significantly prolonged as well. As a result of ultra-low-dose aspirin injection, both alterations have been normalized in both studies. It was clear that the laser-induced thrombus production was a new, interesting, *in vivo* model for observing these alterations in portal hypertension. Ultra-low-dose aspirin was not only normalizing these platelet-endothelial cell interaction alterations but was normalizing the induced hemorrhagic time as well. Further research was aimed to clarify the mechanism underlying these effects.

### 3.2. Second Study

#### 3.2.1. Inhibition of NO Synthesis or Inhibition of COX and Its Modifications of the Normalizing Effects of Ultra-Low-Dose Aspirin in Experimental Prehepatic Portal Hypertension

As nitric oxide (NO) and prostacyclin (PGI_2_) are two major endothelial vasodilators that play a major role in the pathophysiology of portal hypertension [20] and both decrease platelet aggregation, inhibition of their effects or function were tried in the search of an explanation in the mechanism of effect of ultra-low-dose aspirin. Besides, NO and PGI_2_ synthesis are modified by aspirin [21]. A new study was then designed with 12 groups of rats. The first 4 groups were identical to the previously described study and were used to confirm the previous results and as baseline for the study. The other 8 groups were also sham and portal hypertensive rats with and without ultra-low-dose aspirin, but 4 of them received L-nitro-arginin methyl Ester (NAME, an inhibitor of NO synthesis) and the other 4 indomethacin, a nonselective COX inhibitor [22]. The NAME group has not shown clear modifications in the normalizing effect of ultra-low-dose aspirin. On the contrary, indomethacin increased the antithrombotic changes observed in portal hypertensive animals and induced an antithrombotic effect in sham-operated rats as well. Despite this antithrombotic effect, the prothrombotic effect of ultra-low-dose aspirin also increased in sham-operated as well as in portal hypertensive group. Indomethacin produced also a prolonged Induced hemorrhagic time that was normalized by ultra-low-dose aspirin in sham-operated rats but was not modified in portal hypertensive ones. Indomethacin had an antithrombotic effect on the rat but increased the prothrombotic effect of ultra-low-dose aspirin. This paradoxical effect was supposed to be caused by the differential effect over COX 1 and COX 2.

### 3.3. Third Study

#### 3.3.1. Effects of Previous Inhibition of COX 1 or COX 2 on the Prothrombotic Effects of Ultra-Low-Dose Aspirin

To clarify the apparently opposed effects of indomethacin in portal hypertensive rats treated with ultra-low-dose aspirin, a new study was designed [23]. In this study, 3 groups (sham placebo-portal hypertension, placebo-portal hypertension, ULDA) were used as control. Other two subsets of 3 groups with the same above described treatments and surgery were treated with SC 560 (a selective COX 1 inhibitor) or NS 398 (a selective COX 2 inhibitor) previous to the treatment with ultra-low-dose aspirin. The treatment with selective COX 1 inhibitor induced a tendency to decrease the production of thrombi in sham-operated animals. Despite this apparently antithrombotic effect, the effect of ultra-low-dose aspirin of increasing the number of emboli in portal hypertensive rats remained equally active. The selective inhibition of COX 2 made the effect of ultra-low-dose aspirin inactive. Dosing 6 PGF1*α* in this study has shown increased values in portal hypertension. The use of ultra-low-dose aspirin returned these values to normal in spite of the presence of portal hypertension. The inhibition of COX 2 reduced the decrease in thrombi production observed in portal hypertensive rats. Beside this prothrombotic effect, similar to the effect of ultra-low-dose aspirin, the pretreatment with COX 2 inhibitor blunted the effect of ultra-low-dose aspirin in thrombi production of portal hypertensive rats. The conclusion of this study stated that ultra low dose aspirin was acting through the COX 2 pathway.

### 3.4. Fourth Study

#### 3.4.1. Effects of Ultra-Low-Dose Aspirin in Rats with Biliary Cirrhosis

**Figure 1:**
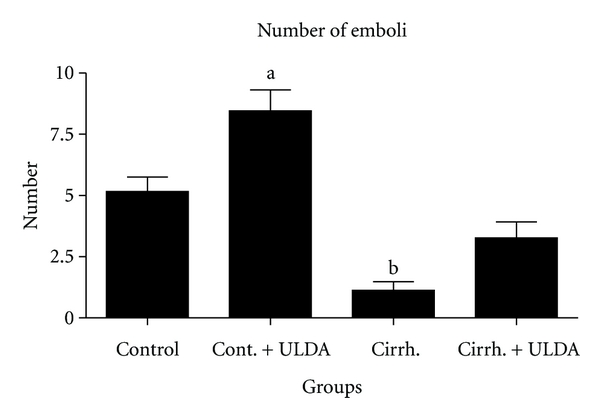
Laser-induced thrombus formation. Study of number of emboli (expressed in number). Control: sham-operated rats. Cont. + ULDA: sham-operated rats pretreated with ULDA. Cirrh.: cirrhotic rats. Cirrh. + ULDA: cirrhotic rats pretreated with ULDA. ^a,b^ *P* < 0.001 versus control (ANOVA, Bonferroni post-test).

**Figure 2:**
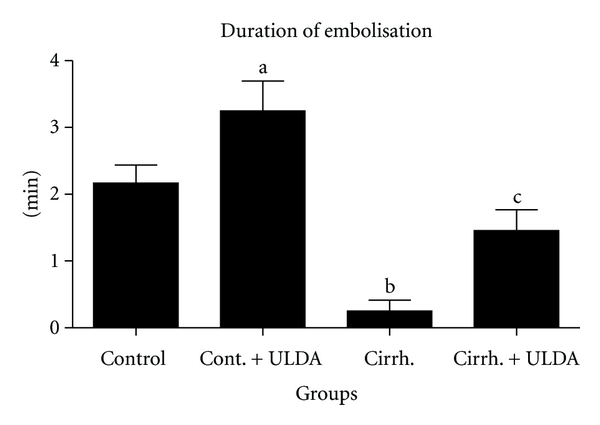
Laser-induced thrombus formation. Study of Duration of embolisation (expressed in minutes). Control: sham-operated rats. Cont. + ULDA: sham-operated rats pretreated with ULDA. Cirrh.: cirrhotic rats. Cirrh + ULDA: cirrhotic rats pretreated with ULDA. ^a^ *P* < 0.05 versus control; ^b^ *P* < 0.01 versus control; ^c^ *P* < 0.05 versus cirrhosis (ANOVA, Bonferroni post-test).

**Figure 3:**
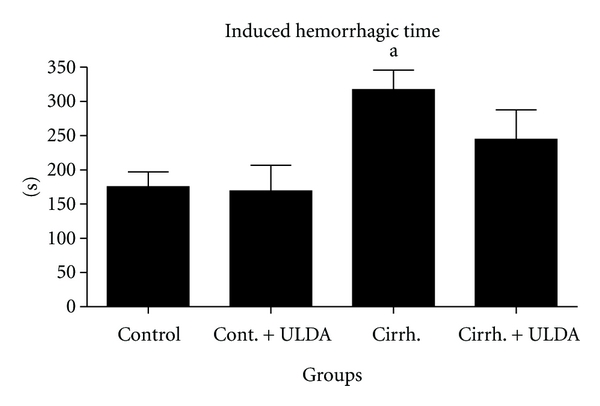
Study of induced hemorrhagic time (expressed in seconds). Control: Sham operated rats. Cont + ULDA: Sham-operated rats pretreated with ULDA. Cirrh.: Cirrhotic rats. Cirrh. + ULDA: Cirrhotic rats pretreated with ULDA. ^a^ *P* < 0.05 versus Control. (ANOVA, Bonferroni post-test).

**Figure 4:**
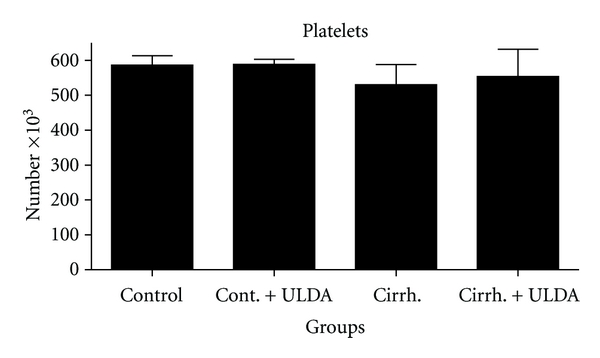
Study of platelet count (expressed in number × 10^3^). Control: sham-operated rats. Cont + ULDA: Sham-operated rats pretreated with ULDA. Cirrh.: cirrhotic rats. Cirrh. + ULDA: cirrhotic rats pretreated with ULDA.

**Figure 5:**
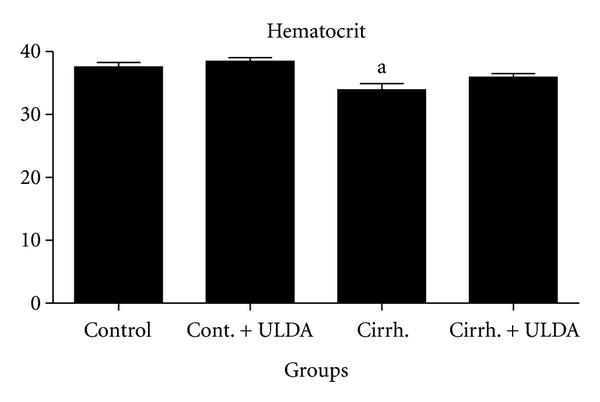
Study of Hematocrit (expressed in %): Control: Sham operated rats. Cont + ULDA: Sham operated rats pretreated with ULDA. Cirrh: Cirrhotic rats. Cirrh + ULDA: Cirrhotic rats pretreated with ULDA. ^a^ *P* < 0.01 versus Control. (ANOVA, Bonferroni post-test).

Rats with prehepatic portal hypertension have an almost normal liver. This experimental model was chosen to focus the attention on the effects of portal hypertension on thrombi formation and interaction between platelet and endothelial cell. In an unpublished study of our laboratory, the effect of ultra-low-dose aspirin was tested in cirrhotic rats with ascites produced by 30 days of common bile duct ligation. Although patients with primary biliary cirrhosis (PBC) showed better preservation of hemostasis with less fibrinolytic activation and platelet function differs between patients with cholestatic and noncholestatic liver disease and is stable or even hyperactive in patients with PBC and primary sclerosing cholangitis [24, 25], common bile duct ligated rats have shown a clear decrease in thrombi formation in the laser study and a prolonged induced hemorrhage time. Hemorrhage can be a complication of biliary cirrhosis. For example, PBC patients had an earlier recurrence of esophageal varices compared to non-PBC patients and variceal bleeding complicates PBC, when it is histologically advanced [26, 27]. In our study with laser-induced thrombosis, 54 rats were randomly assigned to 4 groups, two of them underwent common bile duct ligation and in the other two bile duct was identified but not ligated. One group of the sham-operated rats and one of the groups with biliary cirrhosis were treated with ultra-low-dose aspirin; the other two received placebo. After 30 days of common bile duct ligation induced a decreased thrombi formation and a decreased time of embolization (Figures 1 and 2). Induced hemorrhagic time was clearly prolonged (Figure 3). After treatment with ultra-low-dose aspirin, sham-operated rats increased the number of emboli and the duration of embolization and the rats with biliary cirrhosis normalized thrombosis and hemorrhage. Statistical results are shown in Figures 1 to 3.  Figure 4 shows that the platelet number remained stable in all the 4 groups. Only a small hematocrit drop was observed in the group with biliary cirrhosis and treated with placebo (Figure 5).

## 4. General Discussion

These studies with the laser-induced thrombosis model have shown that this *in vivo* model seems to be useful in the direct observation of the interaction between the platelets and the endothelial wall. This interaction is clearly modified in both experimental models of portal hypertension. Although laser-induced thrombi generation alterations are accompanied by a prolongation of induced hemorrhagic time, they do not always move in a parallel way. This model proves itself as a very sensitive indicator of alterations in the platelet-endothelial cell interaction. The direct observation of the mesenteric vascular bed keeps interference with the haemostatic process to a minimum and does not use perfusion chambers, perfusion pumps, or anticoagulated blood.

The effects of ultra-low-dose aspirin have shown an increased thrombi generation in the normal rat. This effect appears to normalize the decreased thrombi formation observed in portal hypertension. 

The use of two different models of portal hypertension, one with an almost normal liver and the other with a clear cirrhosis, ascitis, and edema, shows that the prothrombotic effect of ultra-low-dose aspirin acts regardless of the liver function. 

Regarding hemorrhage, the same normalizing effects of these doses of aspirin have been observed in laser-induced thrombosis and in induced hemorrhage time. 

The studies with indomethacin and with selective COX inhibitors show that this effect seems to act through the COX 2 pathway. This observation has been confirmed in a later study in COX 1−/− or COX 2−/− knockout mice [28]. COX 1 selective inhibition has an effect opposite to that of ultra-low-dose aspirin whether COX 2 selective inhibition induces a prothrombotic effect similar to the effect of ultra-low-dose aspirin and decreases the effect of a posterior administration of ultra-low-dose aspirin. 

This ultra-low dose of aspirin offers the possibility of a new approach to the treatment of the hemorrhagic tendency in patients with portal hypertension, not centered in hemodynamic factors but on normalizing the interaction between platelet and the endothelial cell. 

In conclusion this paper reviews the prothrombotic properties of ultra-low-dose aspirin in prehepatic portal hypertensive rats and in bile- duct-ligated cirrhotic rats, leading to the normalization of altered thrombi formation in the mesenteric vascular bed and the normalization of induced hemorrhagic time. These beneficial effects could be due to COX 2 inhibition and could be useful in the treatment of the altered primary haemostasis observed in this pathology and in the prevention of hemorrhagic complications of these patients.
